# Tobacco Products, Periodontal Health and Education Level: Cohort Study from Sweden

**DOI:** 10.3390/dj8030090

**Published:** 2020-08-10

**Authors:** Anna Julkunen-Iivari, Anna Maria Heikkinen, Ismo T. Räisänen, Hellevi Ruokonen, Jukka H. Meurman, Sanna Toppila-Salmi, Per-Östen Söder, Birgitta Söder

**Affiliations:** 1 Department of Oral and Maxillofacial Diseases, University of Helsinki, 00290 Helsinki, Finland; anna.m.heikkinen@helsinki.fi (A.M.H.); ismo.raisanen@helsinki.fi (I.T.R.); 2 Head and Neck Center, Department of Oral and Maxillofacial Diseases, University Hospital, 00014 Helsinki, Finland; hellevi.ruokonen@hus.fi (H.R.); jukka.meurman@helsinki.fi (J.H.M.); 3 Haartman Institute, Medicum, University of Helsinki, 00290 Helsinki, Finland; sanna.salmi@helsinki.fi; 4 Skin and Allergy Hospital, Helsinki University Hospital and University of Helsinki, 00250 Helsinki, Finland; 5 Department of Dental Medicine, Karolinska Institutet, 17177 Stockholm, Sweden; perostensoder@gmail.com (P.-Ö.S.); birgitta.soder@ki.se (B.S.)

**Keywords:** epidemiology, oral health, periodontium, smokeless tobacco, smoking

## Abstract

Background: The aim of this study is to investigate if using tobacco products (including snuff, smoking tobacco and dual-using) associates with periodontal health, education level and mortality in a Swedish cohort, hypothesizing that tobacco products affect periodontal health, associate with lower education and increase the risk of death. Method: Study cohort of 1080 subjects aged 31–40 years (528 men, 552 women) was clinically examined and interviewed in 1985 and followed for mortality until 2015. Subjects were classified into two groups: “tobacco users” and “non-users”. Associations between periodontal health parameters, tobacco products, education level and age of death were analysed. SPSS was used for analyses. Results: Tobacco products, as well as education level associated, with poor periodontal health. Tobacco users and lower education was linked to higher plaque-, calculus- and gingival-index scores than non-users (*p <* 0.001). They also had significantly higher prevalence of deep periodontal pockets (≥5 mm) (*p <* 0.001 and 0.010, respectively), missing teeth (*p =* 0.010 and 0.003, respectively) and lower education level (*p* < 0.001) compared with non-users. However, tobacco product users did not die significantly earlier than non-users. Conclusion: Tobacco products had a negative impact on periodontal health. Tobacco product users were less educated. However, using tobacco products may not cause premature death.

## 1. Introduction

Periodontal health is a state free from inflammatory periodontal disease, where subgingival microbial organisms coexist in a harmony. Clinically, periodontal health can be determined as lack of histological or anatomical evidence of periodontal inflammation in periodontium. When the balance of microbiota is disturbed, the host immune and inflammatory response induces progression of periodontal disease [1], which can lead to a chronic periodontitis, where pocket regression is one of the signs [2]. Environmental factors such as smoking could also affect periodontal disease progression as a grade modifier [3,4,5]. Smoking has proven to be a critical risk factor for chronic periodontitis both in adolescents and in adults [5,6,7]. In addition, smoking tobacco increases the risk for many other oral health problems, including oral cancer, coronary heart disease and mortality [7,8].

Snuff users are subjected to the same amount of nicotine than smokers get from cigarettes [9]. The use of snuff (Swedish moist snuff) may be associated with localized gingival recession, leukoplakia and clinical attachment loss [9,10]. However, some researchers have reported that snuff use is not a risk for periodontal diseases [10,11]. Negative health effects, such as increased risk for cancer, ischaemic heart disease and diabetes type II, are less known from studies with snuff users than those from smokers [11,12,13]. Lower socioeconomic position (i.e., education, income and poverty-income ratio) associates with poorer periodontal health status [14,15]. Almerich-Silla et al. (2017) found in their cross-sectional study a statistically significant relationship between low educational level, low social class and a higher prevalence of deepened periodontal pockets in the adult population [16].

In a recent study, current snuff users showed higher mortality risk for coronary heart disease relative to those who never had used tobacco products [17]. In another study, daily use of tobacco products did increase the risk for mortality [18]. The authors found that current smoking is associated strongly with chronic lower respiratory disease, while current smokeless tobacco users had increased mortality risk for heart disease and all cancers [18].

With this background, the aim of the present study was to investigate associations between the use of tobacco products, education level and periodontal health, and furthermore their relation to mortality, in our long-term follow-up cohort study. We hypothesized that users of tobacco products have poorer periodontal health, lower education level and increased risk of death than non-users.

## 2. Materials and Methods

The study subjects’ inclusion criteria were that they all were born on the 20th day of any month in 1945 to 1954, they were current users or non-users of the tobacco products, and they were inhabitants of the Stockholm area. The study subjects were aged between 31 and 40 years (mean age 35.7 years) at the baseline in 1985. Altogether, 3273 randomized subjects were invited to the study; 1597 subjects did not participate. In total, 1080 subjects were included to the study (528 men and 552 women). Exclusion criteria was that subjects were born in any other day of the month than 20th, they were aged other than 31 to 40 years at the baseline or subjects were ex-users of tobacco products. The study cohort was examined clinically at baseline in 1985 and their mortality have been followed up to 2015. Clinical examinations were made by six periodontists in the Stockholm area in 15 community dental centres. All subjects provided ethics form of consent to use their data for analysis in writing. Data from the Swedish National Death Register that list the cumulated causes of all deaths were used in the analyses. In the study cohort, 138 subjects had passed away during the follow-up by 2015. The World Health Organization International Classification of Diseases -7-9-10 diagnoses were extracted from the National Hospital Registers of Sweden.

Periodontal health parameters were recorded at baseline, and these were then analysed regarding the reported tobacco using habits. The subjects informed in writing their tobacco using habits and if they were ex-users, current users or non-users of the tobacco products. The amount of the tobacco products in daily use was not informed. The age of death of the patients was recorded and analysed between the groups.

According to the 2017 World Workshop Classification for Periodontal Disease, periodontitis is classified based on staging by clinical attachment (CAL) loss as stage I (1–2 mm), stage II (3–4 mm) and stage III and IV (≥5 mm) [3]. If this is not available, then radiographic bone loss (RBL) should be used. Grade modifiers, such as diabetes and smoking should be used as indicators of the rate of periodontitis progression [3,19]. However, the new classification for periodontal disease could not be used in our study, because of the lack of CAL measurements, as well as bone loss, assessed from radiographs. This study was collected in 1985, including periodontal examination and recording periodontal pocket depths (PD), dental plaque index (PI), gingival index (GI) and calculus index (CI) [20], as well as recording the number of teeth and number of missing teeth as well [20,21]. Periodontal pocket depths were measured from all teeth and from six surfaces with a Hu-Friedy (PCPUNC 15) periodontal probe (Hu-Friedy, Chicago, Il, USA) and pockets ≥5 mm were considered deep and used accordingly in the statistical analyses [21]. Radiological examinations were not taken systematically from all patients. Details of this cohort study have been published earlier [22,23].

We included subjects with different habits of snuff use and smoking tobacco and categorized them into two different groups, compared with each other. Group 1, “tobacco users”, included current smokers and snuffers. Since this is a retrospective study, the number of cigarettes per day could not be found in each patient. Group 2, “non-users”, included non-snuffers and non-smokers. We analysed cross-sectional associations between the subjects in the groups with the following variables: sex (male/female), working status (employed/unemployed), education (compulsory school/be of higher education), income, marital status (single/married/divorced), dental visits once a year (yes/no), periodontal pockets (≥5 mm) (yes/no), PI-, GI-, CI- scores, missing teeth (yes/no) and age in the beginning of the study in 1985.

Statistical analyses were made with the SPSS version 25.0 (IBM SPSS Statistics for Windows, IBM Corp., Armonk, NY, USA). Cross-tabulation with Fisher’s exact test was used for testing differences between the proportions of the two groups for statistically significant results. Mann–Whitney U test was used for testing if there was a difference in PI-, CI- and GI- scores between the sums of ranks of the two groups. The significant difference in PI-, CI- and GI- scores was confirmed by *t*-test. When stratified for current tobacco product user status and education level, pairwise post hoc comparisons were calculated by Dunn–Bonferroni test. Comparison of the difference in age and income between the two groups of patients was made by t-test. Kaplan–Meier survival analysis was used for estimating and graphing the survival probabilities as a function of time, and the log-rank, Breslow and Tarone–Ware tests were used for comparing the survival in the different groups. Statistical significance was set at *p* values ≤ 0.050.

The present study is a human observational study and investigators have conformed to the CONSORT guidelines. No harm or unintended effects were caused to any group. The study had been approved by the Ethics Committee of the Karolinska Institutet and Huddinge University Hospital in Sweden (Dnr 101/85 and revised in 2012/590-32). The study was conducted in accordance to the Declaration of Helsinki.

## 3. Results

Patient characteristics are given in Table 1. In this study, there was not a significant difference between the proportions of females and males in current tobacco product users, comprising Swedish moist snuff and smoking tobacco, and non-users (*p* = 0.503). Most of the participants were employed and the employment status did not significantly differ between the groups (*p* = 0.601). Non-users were more likely to be of higher education than current tobacco product users (*p <* 0.001). However, regarding marital status, there were more singles and divorced individuals and less married ones among tobacco product users (*p <* 0.001). Current tobacco product users also had significantly smaller mean income compared with the non-users (*p <* 0.001). Furthermore, they also visited a dentist once a year more often than non-users (*p* = 0.008). See Table 1 for more information about the patient characteristics.

**Figure 1:**
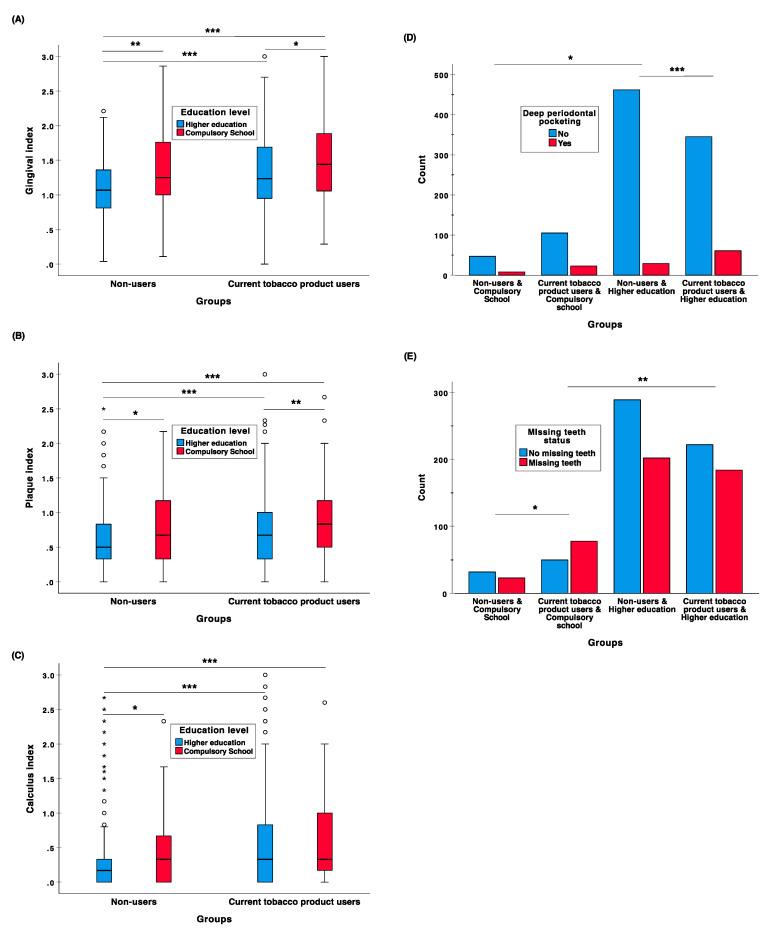
Periodontal health parameters: (**A**) gingival index, (**B**) plaque index, (**C**) calculus index, (**D**) ≥5 mm deep periodontal pocketing and (**E**) missing teeth status among current tobacco product users and non-users categorized by education level (compulsory school and higher education). All significant (* *p <* 0.05; ** *p <* 0.01; *** *p <* 0.001) pairwise post hoc comparisons (Dunn–Bonferroni test for GI-, PI- and CI-scores and Fisher’s exact test for the prevalence of deep periodontal pocketing and missing teeth) are marked in the plots. The box-and-whiskers plots illustrate the median, quartiles, and extreme values.

Periodontal health parameters for current tobacco product users and non-users categorized by their education level are presented in Figure 1. Overall, the median score of PI-, CI- and GI-scores for tobacco product users differed significantly from the respective medians of non-users (*p* < 0.001). Likewise, there was a significant difference between individuals with an education background of compulsory school and higher education according to PI-, CI- and GI-scores (*p* < 0.001). Furthermore, non-users with higher education had significantly smaller median score of PI-, CI- and GI-scores than current tobacco product users or individuals with a compulsory school background (Figure 1A–C). Furthermore, among current tobacco product users, median score of PI-, and GI-scores were significantly higher for individuals with a lower education compared with higher education (Figure 1A,B).

The proportion of tobacco product users with deep periodontal pockets (≥5 mm) (15.7%) was higher than that in non-user group (6.8%) (*p* < 0.001). Similar findings were discovered between individuals with compulsory school and higher education, as the proportions were 16.9% and 10.0%, respectively (*p* = 0.010). Again, respective significant differences between the groups of current tobacco product user status (users 49.1% and non-users 41.2%) and education level status (lower education 55.2% and higher education 43.0%) were also observed in the number of missing teeth (*p* = 0.010 and *p* = 0.003, respectively). See Supplementary Table S1.

When stratified for education level (Figure 1), tobacco product usage was associated with increased prevalence of deep periodontal pockets among higher educated individuals compared to those with lower education (15.0% vs. 5.9%; *p <* 0.001), while for non-users with higher education, the prevalence of deep periodontal pocketing was significantly less common compared with individual with lower education (5.9% vs. 14.5%; *p =* 0.024). Furthermore, missing teeth were more common among individuals with a compulsory school background if they were also current tobacco product users (60.9% vs. 41.8%; *p =* 0.023) (Figure 1D). Similarly, among current tobacco product users, the prevalence of missing teeth was higher in individuals with lower education than with high education (60.9% vs. 45.3%; *p =* 0.002) (Figure 1E).

**Figure 2:**
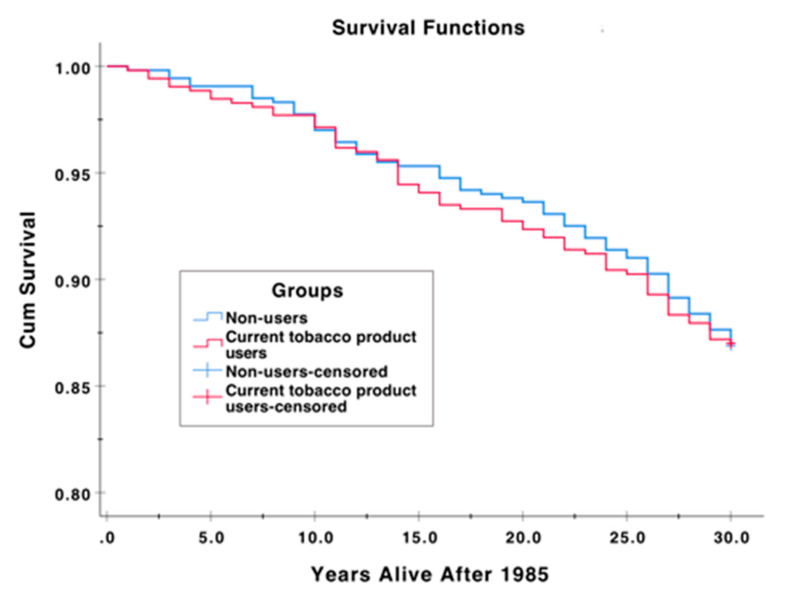
Kaplan–Meier survival analysis of the cumulative survival proportion against time for current tobacco product users and non-users.

Table 2 gives the medians and the interquartile ranges (IQR) for the age of death and the age of patients that had survived by the end of the study; the findings are categorized according to the status of patient’s use of tobacco products. Figure 2 and Table 2 shows the results from the survival analysis. Overall, mortality rates were low for both tobacco product users and non-users (the numbers of individuals who died were 68 and 70, respectively). Furthermore, there was not a significant difference between these groups in survival time (Table 2). Table 3 gives ICD-7-9-10 codes of the disease diagnoses among the individuals who had died during the follow-up by 2015. Current tobacco users had more cancer (C155, C229, C240, C249, C450, C509, C549, C619, C669, C679, C739) and gastroenterology diagnoses (K269, K439, K572, K823, K830, K838) compared to non-users. Both groups had same amount of diagnosed cardiovascular diseases.

## 4. Discussion

In the present study, using tobacco products (Swedish moist snuff, smoking tobacco and dual-using) associated with poor periodontal health as well as with low education. However, no association between mortality and using tobacco products could be found. In this respect, our hypothesis was rejected.

Tobacco product users showed poorer periodontal health with higher prevalence of deep periodontal pockets, higher PI-, GI- and CI-scores than non-users [24]. Nicotine and other compounds in snuff and cigarette may cause upregulation of pro-inflammatory cytokines and inflammatory mediators affecting the tooth supporting tissues thus leading to periodontal disease [25]. In the present study, tobacco users had significantly more missing teeth compared to non-users. This may also be a sign of earlier periodontal disease. In previous studies, periodontitis had been suggested as the most common cause of tooth loss in patients over 40 years [26] and missing teeth have been proposed as a surrogate marker for periodontal disease [26,27,28].

Lower education level associated with poorer periodontal health in the present study. This finding is in line with previous studies [15,29]. In their review study, Borrell and Crawford (2012) evaluated commonly used socioeconomic position indicators (i.e., income, education level, poverty-income ratio) and periodontitis from periodontal epidemiology studies. The data from the National Health and Nutrition Examination Survey 1999–2004 was used and results showed that individuals (aged 20–64 years) with less than a high school education had almost three times higher prevalence of periodontitis compared to persons with more than a high school education (17.3% vs. 5.8%). The results were similar with individuals aged ≥65 years (16.6% vs. 8.3%). In their conclusion, persons with lower socioeconomic status have poorer periodontal health [15].

Use of tobacco products is more common among subjects with lower education level [30]. This was also confirmed by our present findings. In a study from Sweden, comprising 92,563 subjects, of whom 14,373 were snuff users and 10,205 were current or previous smokers, men with low education level were more often snuff users, but in women the opposite was found. Women with higher education level in fact used more often snuff than those with low education level, and the middle-aged seemed to increase their use of snuff, especially if they were previous or current smokers [30].

In the present study tobacco product users did not die earlier than non-users. This was in contrast to our expectation. In other 12-year and 18-year follow-up studies, respectively, subjects using snuff or chewing tobacco had higher death rates than non-users. The main conclusion by these authors was that current use of snuff or chewing tobacco may increase mortality linked to heart disease and stroke [31]. In our findings, amount of diagnosed cardiovascular diseases were equally the same in both groups. Tobacco product users had more diagnosed cancer and gastroenterology diseases compared to non-users, which is in line with other studies [8,32].

The strength of our study is that it is based on comprehensive patient data, national and reliable mortality records, and a long follow-up of 30 years. In addition, periodontal pockets had been measured from all teeth, not only from index teeth. The clinical relevance of the present study supports the previous knowledge that use of tobacco products (including both smokeless and smoking tobacco) is associated with poorer oral health, as well as with lower education level. This finding supports the importance of taking account prerequisite patient data in in oral health care clinics. The limitation of our study was the absence of knowledge of pack years of tobacco products. In 1985, it was not common to ask about alcohol consumption because that time in Sweden such a question was considered to be as a personal one. This is also another limitation of this study. The study data did not include intra- or extraoral radiographs systematically from the study patients, which is also a limitation.

In the present cohort, the oldest participants alive were 70 years old by the year 2015. The mean life expectancy in Sweden is around 80 years. Hence, deaths before this age can be regarded as premature deaths. Finally, this study was cross-sectional, as patients were examined and interviewed only at the beginning of the follow-up period, namely in the year 1985. Oral health data and use of tobacco products were not available from the years 1985–2015. Consequently, our results represent the role of anamnestic data on the outcomes.

## 5. Conclusions

Our results partly confirmed our study hypothesis by showing that use of tobacco products associated with lower education level and higher prevalence of deep periodontal pockets, together with the other periodontal health parameters recorded. However, contrary to what we had expected, compared with non-users, tobacco users did not seem to die earlier.

## Figures and Tables

**Table 1:** Patient characteristics from 1080 subjects.

	Current Tobacco Product Users	Non-Users	Total	*p* Value
	N = 534	N = 546	N = 1080	
**Sex N (%)**				0.503
Male	267 (50.0)	261 (47.8)	528 (48.9)	
Female	267 (50.0)	285 (52.2)	552 (51.1)	
Total	534 (100.0)	546 (100.0)	1080 (100.0)	
Age (Mean, SD)	35.96 (2.83)	35.44 (2.86)	35.70 (2.86)	0.004
**Working status N (%)**				0.601
Employed	481 (90.2)	498 (91.2)	979 (90.6)	
Unemployed	52 (9.8)	48 (8.8)	100 (9.4)	
Total	533 (100.0)	546 (100.0)	1079 (100)	
**Education N (%)**				<0.001
Compulsory School	128 (24.0)	55 (10.1)	183 (16.9)	
Higher education	406 (76.0)	491 (89.9)	897 (83.1)	
Total	534 (100.0)	546 (100.0)	1080 (100.0)	
**Marital status N (%)**				<0.001
Single	211 (40.7)	174 (32.8)	385 (36.7)	
Married ^1^	218 (42.0)	298 (56.1)	516 (49.1)	
Divorced ^2^	90 (17.3)	59 (11.1)	149 (14.2)	
Total	519 (100.0)	531 (100.0)	1050 (100)	
**Income (Mean, SD)**	1776.44 (927.10)	1998.32 (1157.62)	1888.65 (1055.38)	<0.001
**Dental visits once a year **				0.01
**N (%)**				
Yes	156 (29.2)	121 (22.2)	277 (25.6)	
No	378 (70.8)	425 (77.8)	803 (0.74)	
Total	534 (100.0)	546 (100.0)	1080 (100.0)	

^1^ include married and common-law married, ^2^ include divorced and widow. *p* values by Mann Whitney U test (Age, Income) and Fisher’s exact test (Sex, Working status, Education, Marital status). *p* values by Fisher’s exact test.

**Table 2:** Medians and interquartile range (IQR) for the age of death and the age of patients that survived past the end of the study categorized according to patient’s tobacco product usage. Overall test of equality of survival distributions for current tobacco product users and non-users.

Groups	Age at the End of the Study	Age of Death	Survival Time	Survival Analysis (Overall Comparisons)	*p* Value
	Median (IQR)	Median (IQR)	Mean (95% CI)	Test	
Current tobacco product users	66.00 (64.00–68.00)	55.00 (46.00–60.00)	28.34 (27.89–28.79)	Log Rank (Mantel-Cox)	0.993
Non-users	65.00 (63.00–68.00)	55.00 (48.50–62.00)	28.52 (28.10–28.94)	Breslow (Generalized Wilcoxon)	0.974
Overall	66.00 (63.00–68.00)	55.00 (47.00–61.75)	28.43 (28.13–28.74)	Tarone-Ware	0.991

IQR: Interquartile range; CI: Confidence interval.

**Table 3:** ICD-7-9-10 codes of the diseases in both groups (current tobacco product users and non-users) of the deceased subjects.

Current Tobacco Product Users ICD Codes	Non-Users of Tobacco Products ICD Codes
162x, 205A, 214X, 234, 296X, 303X, 401.99, 401X, 413X, 427.99, 427A, 427D, 431X, 454X, 585X, 615.2, 969E, 996W, A151, A410, C155, C229, C240, C249, C450, C509, C549, C619, C669, C679, C739, D333, E119, I120, I200, I209, I212, K269, K439, K572, K823, K830, K838, M161, R074, R119, R568.	233.99, 428X, 428X, 450.01, 454.99,456B, 493X, A469, C509, C509, C509, C787, F019, F101, G408, G473, G479, I209, I214, I269, I420, I420, I471, I509, I509, I802, I803, I872, K253, K620, M165, R060, R061, R519, R568.

## Supplementary Materials

The following are available online at https://www.mdpi.com/2304-6767/8/3/90/s1.
