# Supplementary material for: Tobacco Products, Periodontal Health and Education Level: Cohort Study from Sweden
Source: Dent J (Basel). 2020 Aug 10;8(3):90. doi: 10.3390/dj8030090 (PMC7559463; doi:10.3390/dj8030090)
Supplement: Supplementary file 1 [file dentistry-08-00090-s001.pdf]

dentistry journal

MDPI

Article

Tobacco Products, Periodontal Health and Education Level: Cohort Study from Sweden

Anna Julkunen-Iivari [1,2,*], Anna Maria Heikkinen [1,2], Ismo T. Räisänen [1], Hellevi Ruokonen [2], Jukka H. Meurman [2], Sanna Toppila-Salmi [3,4], Per-Östen Söder [5] and Birgitta Söder [5]

[1] Department of Oral and Maxillofacial Diseases, University of Helsinki, 00290 Helsinki, Finland; anna.m.heikkinen@helsinki.fi (A.M.H.); ismo.raisanen@helsinki.fi (I.T.R.)
[2] Head and Neck Center, Department of Oral and Maxillofacial Diseases, 00014 Helsinki, University Hospital, Helsinki, Finland; hellevi.ruokonen@hus.fi (H.R.); jukka.meurman@helsinki.fi (J.H.M.)
[3] Haartman Institute, Medicum, University of Helsinki, 00290 Helsinki, Finland; sanna.salmi@helsinki.fi
[4] Skin and Allergy Hospital, Helsinki University Hospital and University of Helsinki, 00250 Helsinki, Finland
[5] Department of Dental Medicine, Karolinska Institutet, 17177 Stockholm, Sweden; perostensoder@gmail.com (P.-Ö.S.); birgitta.soder@ki.se (B.S.)
* Correspondence: anna.julkunen@helsinki.fi; Tel.: +358-456-783-186

Supplementary Materials:

Table S1. Periodontal findings and missing teeth recorded of the patients.

	Current Tobacco Product Users N = 534	Non-users N = 546	*P* Value	Compulsory School	Higher Education	*P* Value
Plaque index Median (IQR)	0.67 (0.67)*	0.50 (0.50)+	< .001	0.83 (0.67)	0.67 (0.67)	< .001
Calculus index Median (IQR)	0.33 (0.83)*	0.17 (0.42)+	< .001	0.33 (0.75)	0.17 (0.50)	< .001
Gingival index Median (IQR)	1.29 (0.79)*	1.07 (0.57)+	< .001	1.39 (0.81)	1.11 (0.62)	< .001
Periodontal pockets (≥5 mm) N (%)			< .001			0.010
Yes	84 (15.7)	37 (6.8)		152 (83.1)	807 (90.0)	
No	450 (84.3)	509 (93.2)		31 (16.9)	90 (10.0)	
Missing teeth N (%)°			.010			0.003
Yes	262 (49.1)	225 (41.2)		101 (55.2)	386 (43.0)	
No	272 (50.9)	321 (58.8)		82 (44.8)	511 (57.0)	

P values by Mann–Whitney U test (plaque index, calculus index, gingival index) and Fisher's exact test (diagnosed periodontitis, periodontal pockets, missing teeth)
* No data from 2 patients
+ No data from 1 patients
° Reason for tooth loss is unknown
